# Assessing vicarious trauma and attitudes toward mental health services for forensic sciences professionals

**DOI:** 10.1111/1556-4029.70244

**Published:** 2025-12-04

**Authors:** Kathryn C. Seigfried‐Spellar, Sonali Tyagi

**Affiliations:** ^1^ School of Criminal Justice Michigan State University East Lansing Michigan USA; ^2^ Department of Computer & Information Technology Purdue University West Lafayette Indiana USA

**Keywords:** forensic science professionals, mental health, needs assessment, psychological distress, vicarious trauma

## Abstract

Research indicates that forensic science professionals operate under significant pressure, the magnitude of which varies depending on their field, workload, case type, tenure, and the evidentiary significance of their testimony in court. This study conducted a needs analysis of forensic science professionals by examining their psychological well‐being, coping mechanisms, and barriers to accessing mental health services. A total of 618 individuals from the AAFS community completed the anonymous online Qualtrics survey. Out of 618 participants, 601 participants responded to the PCL‐5 questionnaire, which revealed that 8.4% (*n* = 52) of the 601 forensic science professionals have probable PTSD symptoms. There was no significant difference between genders for probable PTSD symptoms. However, 76.5% of the probable PTSD symptomatic population were more likely to seek therapy as a result of work‐related stress. Female forensic science professionals and forensic science professionals with fewer years of work experience were likely to report more psychological distress symptoms. A similar pattern was noticed with using coping strategies and experiencing mental health barriers based on gender and years of working experience. In general, forensic science professionals were more likely to report positive coping strategies (e.g., talking with a spouse) and less likely to use maladaptive strategies (e.g., drugs). Finally, female forensic science professionals reported 5 out of 14 mental health barriers to help‐seeking behavior. Findings support the need to implement a supportive organizational culture, including training, awareness programs, and encouraging help‐seeking behaviors, irrespective of gender and years of experience.

**Highlights**

- Female forensic science professionals experienced more psychological symptoms than their male counterparts.
- Forensic science professionals with fewer years of work experience reported more psychological symptoms.
- Counseling was required for only a few participants as part of their job position.
- Female forensic science professionals reported more mental health stigmas and barriers than males.
- Forensic science professionals with fewer years of work experience reported more mental health stigmas and barriers.

## 1 INTRODUCTION

Forensic science is the use of the scientific method or related skills to investigate crime and analyze legal evidence [1]. Forensic scientists typically use their expertise to help members of the legal system understand scientific evidence, often taking the role of expert witnesses in court cases [2]. There are approximately 18,600 forensic professionals employed within the United States, with an expected 14% increase by 2033 [3]. The American Academy of Forensic Sciences recognizes twelve forensic disciplines: anthropology, criminalistics, digital and multimedia sciences, engineering and applied sciences, forensic nursing science, general, jurisprudence, odontology, pathology/biology, psychiatry and behavioral science, questioned documents, and toxicology [2]. Forensic anthropology, nursing, odontology, pathology, psychiatry, and toxicology involve the application of medical knowledge to provide an opinion on a range of medicolegal situations. Criminalistics applies the scientific method to analyze physical evidence. Digital and multimedia forensic professionals conduct digital forensic examinations, while forensic engineers employ engineering techniques to determine fault in engineering accidents, environmental damage, and product failures. Finally, the discipline of questioned documents refers to examining documents to verify their authenticity [2]. Each of these disciplines interacts closely with the criminal justice field to investigate crimes.

Research consistently shows that many criminal justice professionals experience work‐related stress that results in vicarious trauma as a result of the investigative process [4]. Despite forensic scientists' prominent role in this process, the majority of research has focused on law enforcement officers investigating homicides, crimes against children, and other violent crimes [4]. Forensic scientists frequently come across traumatic content in the form of graphic crime scene photographs or digital material, physical evidence of assault, or torture. For example, digital forensic examiners are exposed to child exploitation while investigating internet crimes against children (ICAC; [5]). This exposure is multiplied by the sheer number of cases a forensic scientist takes on within a year [6]. Thus, the aim of this study was to assess the psychological well‐being, coping mechanisms, and barriers to help‐seeking for forensic science professionals within the American Academy of Forensic Sciences.

## 2 LITERATURE REVIEW

### 2.1 Forensic science professionals

Many forensic science professionals work under significant pressure; however, the intensity of this pressure varies by their specialty, the nature of their casework, their experience, and the legal significance of the findings they are required to report [7]. Recent studies have found that forensic professionals are at risk for burnout [8, 9, 10, 11], posttraumatic stress disorder (PTSD) [6, 11, 12], and secondary traumatic stress (STS) [8, 9, 10, 11]. PTSD commonly arises from directly experiencing a traumatic event, whereas STS results from indirect trauma, which means being exposed to detailed knowledge of another person's traumatic experience [4]. The sources of stressors among forensic professionals may include organizational stressors (e.g., pressure from supervisors; [13, 14]), occupational stressors (e.g., high demands, unrealistic expectations, and heavy caseload; [15, 16]), and personal stressors (e.g., struggling to balance work with family or personal life; [17]). For example, Schiro et al. [12] found that approximately 74% of forensic practitioners reported at least one work‐related trauma exposure, with provisional PTSD rates nearly twice as high for field‐based examiners (29%) compared to non‐field staff (14.5%). Another study highlighted that forensic scene investigators perceived organizational culture, undervaluation, and inconsistent processes as more psychologically damaging than direct exposure to crime scenes [18].

This is particularly relevant given their exposure to traumatic events, with a study conducted by Lombardo et al. [17] noting that while direct exposure to traumatic case material may not be a primary source of stress for all examiners, burnout and stress are significant issues. They synthesized findings from multiple studies and concluded that burnout and stress are pervasive across forensic roles, including autopsy technicians, forensic physicians, and sexual assault nurse examiners [17]. Despite the emotional demands of the profession, many forensic professionals lack access to adequate mental health resources [8, 17, 19]. Goldstein and Alesbury [8] found that 74% of forensic professionals reported that mental health issues were not adequately addressed during workplace training or academic education. Moreover, although the impact of stressors in the forensic sciences field is well understood, mental well‐being is often considered a taboo subject in this field [7]. Forensic professionals often internalize the belief that seeking help is a sign of weakness, especially in law enforcement‐adjacent roles [11]. They usually worry that admitting to STS or burnout could harm their reputation, limit future promotions, or undermine their credibility in court, leading them to avoid seeking counseling [10]. These mental health stigmas associated with the forensic professions may contribute to feelings of alienation [6].

Coping skills play a crucial role in helping individuals manage stress and shape their approach to handling difficult or high‐pressure situations in the workplace [20]. Moreover, when an individual encounters an event that cannot be changed, they are more likely to rely on emotion‐focused coping strategies [5]. Coping strategies can be adaptive (e.g., talking with others; [21]) or maladaptive (e.g., alcohol or drug use; [22]), and may vary widely among different forensic disciplines. For example, Kelty and Gordon [20] found that high‐performing crime scene examiners employed adaptive mechanisms, such as using black humor, maintaining a work‐life balance, and engaging in peer debriefing, to manage stress. Goldstein and Alesbury [8] highlighted that wellness programs involving therapy dogs, meditation, and group processing were effective in certain organizations. Moreover, organizational factors such as peer support and compensation satisfaction were found to mitigate STS and burnout [6, 10].

### 2.2 Forensic anthropology

Forensic anthropology is a distinct scientific discipline that includes cultural anthropology, archaeology, linguistics, and physical anthropology (human skeletal interpretation) [2, 23, 24]. Forensic anthropology has expanded from its early emphasis on identifying unknown skeletal remains to a broader discipline that integrates archaeological recovery, taphonomy (the study of postmortem alterations to human remains), trauma interpretation, and collaboration within medicolegal systems [23, 25]. They also play a crucial role in disaster victim identification, including identifying and recovering heavily fragmented remains, as well as sorting commingled remains [26, 27, 28]. Moreover, forensic anthropologists employed at medical examiners'/coroners' (ME/C) offices are responsible for managing long‐term unidentified decedent cases, facilitating disposition of unclaimed decedents, submitting and tracking DNA samples, and coordinating mass disaster protocols [29].

The number of cases that forensic anthropologists handle varies significantly depending on their employment type. For example, forensic anthropologists working in applied settings, such as with the Defense POW/MIA Accounting Agency (DPAA) or ME/C offices, tend to have higher caseloads as compared to working in academic settings [30]. Moreover, the demanding nature of work and the challenging environment may result in burnout and stress. A study conducted by Goldstein and Alesbury [8] among forensic practitioners in coroner/medical examiner settings, which included 50% forensic anthropologists, revealed that 64% of respondents felt burnout due to work.

### 2.3 Forensic nursing

Forensic nursing concerns the registered nurse whose primary purpose is to provide medical care for patients who have offended, are likely to offend, or are victims [31]. The inherent nature of the occupation requires everyday exposure to victimized patients, forms of content related to violent incidents, and targeted physical and verbal assault, placing nursing staff at risk of vicarious traumatization and negative emotional and health repercussions [32, 33].

Patients in forensic mental health inpatient facilities frequently pose violence toward nurses, ranging from verbal threats of violence to actual acts resulting in physical injury [34]. For example, Kelly et al. [35] reported that 70% of nursing staff have been physically assaulted in the workplace, sometimes as a consequence of enforcing security‐related restrictions. When non‐restrictive violence prevention and management techniques are insufficient for the safety of patients and staff, physical restraint of a violent patient becomes a necessary procedure used by forensic mental health nurses. Nurses have expressed discomfort with this administration as they experience distress, conflict, and fear concerning it [36]. In addition, there have been reports of nurses consequently obtaining physical injuries while physically restraining a patient, which is also a crucial factor in why they apply for sick leaves [37].

This continuous exposure to patients who have a potential for violence, and the dangerous results it occasionally yields, has planted an element of chronic fear in forensic nursing staff; the long contact time between the staff and the patients allows the former to experience the tension of anticipating violence, eliciting prolonged chronic stress [38]. Similarly, daily exposure to victimized patients and forms of content related to violent incidents places forensic mental health nurses at risk of vicarious traumatization [32]. In a study conducted by Newman et al. [39], one‐fourth of forensic mental health nurses experience high levels of vicarious trauma, supporting the probability of this risk.

Ultimately, these factors have negatively impacted relationships within the forensic setting and contributed to a decrease in job satisfaction [33], resulting from the effects on the emotional well‐being of professionals. Reactions in forensic settings have often been characterized by disinterest, distance, and immobility, particularly when interacting with offenders. These defense mechanisms pose adverse instead of positive effects for professionals and are associated with vicarious trauma [40]. Additionally, forensic nurses experiencing trauma or discomfort in the workplace from exposure to unpleasant incidents or administering physical restraint [36] can perceive the forensic setting as unsafe, subsequently increasing symptoms of burnout and job dissatisfaction and reducing out‐of‐work functioning in the process [32].

However, the managerial and supervising staff, who comprise the forensic environment, also contribute to the occupational stress of nursing staff [38]. In these nursing settings, it is uncommon for fear to be openly discussed, likely due to the reluctance to reveal personal feelings in these environments. This is reflected in a study conducted by Storey and Minto [41], which reported that clinical supervision in secure environments had a low level of acceptance, linking figures such as supervisors to factors of tension. These ultimately indicate low levels of support, which are required from managers and colleagues, to provide confidence and ameliorate the experience of stress in forensic settings [42].

### 2.4 Criminalistics

Criminalist examiners play a critical role in the justice system by applying scientific principles to analyze physical evidence. Their work, as defined, involves the objective application of standard scientific processing techniques such as “biology, chemistry, DNA, mathematics, physics, and firearms/tool marks” to identify, compare, and interpret evidence, ultimately reporting results for use in investigations or trials ([43], para 1).

Significant sources of stress for forensic examiners working in a lab setting include pressure from management and/or supervisors, case backlogs, and the need to process a large number of cases rapidly [10, 44]. Forensic science laboratories, where criminalists primarily work, face a significant problem with case‐file backlogs, which are defined as cases that remain unprocessed or unreported within a specific timeframe [44, 45, 46], especially for forensic biologists and chemists [14, 44, 45]. For example, according to Almazrouei et al. [44], across all forensic examiner categories, forensic biologists reported the highest levels of stress attributed to backlogs (34%), indicating that case backlogs were a primary source of stress.

The constant pressure and complex nature of the work, including the potential for cognitive bias, can influence decision‐making in fields like firearms examinations and DNA analysis [47, 48]. According to the current literature, the challenges related to psychological well‐being are not limited to a single field, as research has examined stress in various disciplines, ranging from toxicology to the psychological impact on DNA analysts [49, 50].

### 2.5 Forensic odontology

Forensic odontology, also known as forensic dentistry, applies dental science to legal investigations. Forensic dentists help identify human remains through dental records, analyze bite marks, estimate age from dental remains, and assess dental injuries in both civil and criminal cases [2]. They may also address dental malpractice concerns, with their work often involving detailed evaluations, written reports, and courtroom testimony [51].

Forensic odontology plays a primary role in identifying when visual recognition or fingerprint analysis is not possible due to postmortem changes, severe trauma, or the absence of usable fingerprint records [52]. Forensic odontology is one of the primary methods for identifying disaster victims [53, 54]. The nature of work for forensic odontologists includes exposure to traumatic scenes [55], decomposed remains [55, 56], violence [52, 56, 57], child abuse cases [52, 56, 57], and disaster victim identification operations [7, 52]. They are expected to operate in psychologically demanding settings, such as mortuaries, and this exposure can lead to various psychological issues, including cognitive bias, PTSD, burnout, and compassion fatigue [7]. Moreover, for forensic odontologists, extended hours of direct exposure to human remains during the identification process are directly related to higher levels of posttraumatic stress symptoms, especially intrusive thoughts [58]. There is limited literature on the psychological well‐being of forensic odontology examiners, or most of the literature is outdated [7].

### 2.6 Digital and multimedia forensics

Digital and multimedia forensic examiners are specialists who investigate digital devices (e.g., computers, phones) and data (e.g., images, audio, video) to find evidence for legal cases [2]. The advancement of digital technology has transformed the criminal investigation process. Due to the proliferation of digital devices, investigators now have access to images and videos of the actual crime itself, in addition to eyewitness accounts, which help them reconstruct the narrative [16, 59]. Therefore, they play a crucial role in identifying and interpreting digital evidence to solve cases because nearly every crime leaves a digital footprint.

Research shows that digital forensic examiners are exposed to high levels of stress due to the nature of their jobs involving distressing material [5, 16, 21, 60, 61] and the volume of digital evidence [16, 62, 63]. Digital forensic examiners and investigators dealing with disturbing media may experience a tremendous amount of emotional burnout [5, 62], anxiety [16], emotional stress [64], physical illness [61, 64], poor psychological well‐being [5, 16, 61], and vicarious trauma (i.e., STS; [5, 21, 61]). Moreover, if a case is particularly unique, the amount of time and frequency of viewing digital media varies [65]. As a result, caseloads are increasing, leading to backlogs and requiring significantly longer than usual to analyze [66].

For digital and multimedia forensic examiners, these stressors may lead to decreased work motivation due to cognitive overload, resulting in reduced innovation and problem‐solving capabilities [16], increased absenteeism [16, 61, 64], and lower productivity [64].

### 2.7 Forensic pathology/biology

A forensic pathologist is a licensed physician practicing as a medical doctor (MD) who has completed additional training and certification in forensic pathology [67], a specialized field of medicine that connects scientific investigation with the legal system [68]. Forensic pathologists apply principles of pathology and medicine to investigate deaths, including those that are sudden, unexplained, or suspicious [68, 69, 70]. Their roles involve regularly examining bodies that are decomposed, dismembered, or violently injured [69, 71, 72] and communicating findings to grieving families, public health authorities, and the criminal justice system [71]. The direct exposure to traumatic visuals may contribute to emotional strain and concerns about infection or contagion [71]. Additionally, some of the most disturbing events for forensic physicians included child victims of trauma, child sexual abuse, and decomposing bodies [19, 73, 74]. Some studies highlighted that forensic medicine professionals, especially autopsy technicians and forensic physicians, are at significant risk of burnout, emotional exhaustion, and show symptoms of PTSD, particularly when handling critical incidents involving children [17, 72, 73].

Additionally, forensic pathologists may be required to interact with the families of the deceased, explaining the cause of death and providing comfort [68, 69, 71], which may increase the risk of vicarious trauma [19] and STS [10]. For example, Brondolo et al. [71] highlighted that direct contact with decedents is a strong predictor of psychological distress among medical examiner personnel, including symptoms of anxiety and emotional numbing when communicating with the decedents' families. Another study conducted by Goldstein and Alesbury [8] found that both direct and indirect contact with grieving families was significantly associated with elevated levels of burnout and occupational stress.

Furthermore, forensic pathologists' findings involve ethical and legal responsibilities, including collaboration with law enforcement during death investigations to provide unbiased medical conclusions about the cause and manner of death [69, 70]. However, they often encounter substantial pressure from external sources, such as political authorities, police agencies, or grieving families, to modify their conclusions, including the cause or manner of death or their courtroom testimony [70]. Moreover, pathologists experience organizational stress when they disagree with supervisors, encounter conflicts with peers, and experience workplace bullying, often due to hierarchical structures in forensic diagnosis [75].

### 2.8 Forensic engineering and applied sciences

The field of forensic engineering is a broad area that encompasses virtually all engineering disciplines applied within a legal setting [76]. Their work frequently involves investigating accidents, product failures, environmental contamination, and criminal incidents [2, 77]. This can include examining building or bridge collapses, automobile crashes, transportation accidents, explosions, shootings, or stabbings [2].

Forensic engineers' duties may vary depending on the context, but they share a common logic of mechanism tracing and compliance with standards. For example, in crane incidents, they audit maintenance and inspection records, analyze wire rope corrosion/fatigue, and evaluate stability envelopes and human factors (e.g., planning and communication; [78]). Similarly, for fire‐damaged concrete, they plan a progressive assessment (including visual inspection, rebound hammer, ultrasonic pulse velocity, and core sampling), interpret the residual capacity, and design proportionate rehabilitation strategies [79]. Forensic engineers and practitioners of applied sciences also work closely with legal systems. They are often called upon in courts to provide expert opinions regarding their examination results in incidents or to offer support in lawsuits filed for negligence, product flaws, or patent infringement [76].

### 2.9 Forensic psychiatry and behavioral science

Psychiatry and behavioral science professionals include forensic psychiatrists, clinical and forensic psychologists, neuropsychologists, and counselors engaged in forensic education, practice, or research [2]. Their work encompasses critical activities, including competency evaluations [80, 81, 82], insanity assessments [80, 81], civil commitment reviews [80], risk assessments [80, 82], analysis of witness statements [82], and trauma‐informed mental health interventions in legal contexts [80]. While their primary forensic role is evaluative, some forensic psychologists and psychiatrists also engage in therapy, which includes providing individual and group therapy to inmates in correctional settings, conducting daily checks on inmates in solitary confinement, and offering crisis counseling [83].

Forensic mental health professionals are exposed to traumatic content, including violent, self‐harming patients, or those who have seriously injured or killed others [84, 85]. Research highlights that repeated exposure to traumatic narratives, crime scene material, and victim testimonies can lead to STS, vicarious trauma, or compassion fatigue among forensic mental health professionals [86, 87, 88]. It is a growing concern, with research indicating high vulnerability among mental health professionals, such as 70% of UK National Health Service (NHS) psychotherapists being susceptible to chronic STS [88]. Due to demanding work conditions, forensic mental health professionals in inpatient and secure settings often face a high risk of burnout, emotional strain, and mental health challenges such as PTSD [84, 85].

Moreover, forensic psychiatrists sometimes have to deal with the victims' suffering and engage in empathetic situations, which may lead to emotional exhaustion [86]. In addition, the adversarial nature of the courtroom can be a significant source of stress for forensic mental health professionals, particularly psychiatrists who are trained to build therapeutic alliances rather than engage in legal confrontation [89]. Similarly, criminal profilers and evaluators dealing with violent or sexual crimes report emotional numbing and disengagement over time [90].

### 2.10 Questioned documents

Questioned document examination (QDE), also known as forensic document examination, is a specialized branch of forensic science primarily used to determine the authorship of signatures and handwriting [2, 91, 92]. However, its scope is much broader, encompassing a comprehensive analysis of the tools used to write, the materials on which they were written, and the devices that produced the documents [2, 93]. Forensic document examiners play a crucial role in legal investigations and judicial proceedings by determining the authenticity, authorship, and integrity of documents, as well as identifying instances of fraud, forgery, and other forms of document alteration [92, 94].

Moreover, examiners can analyze writing instruments and inks to determine links between documents, devices used in their production, and to assess the date of document production using ink analysis and ink dating methods [93, 95]. Forensic examiners not only analyze handwritten documents but also analyze the authenticity, origin, and authorship of typewritten [96] and printed documents [94, 96]. However, unlike other forensic fields, QDE faces ongoing challenges regarding its scientific basis and admissibility in courts, particularly with respect to handwritten documents [97, 98, 99].

### 2.11 Jurisprudence

The Jurisprudence Section refers to forensic science as evidence used by lawyers in presenting cases, the scrutiny of scientific analytical techniques, and the evaluation of forensic science under changing standards of reliability, validity, and admissibility [2]. The Jurisprudence Section of the American Academy of Forensic Sciences (AAFS) facilitates the integration of forensic science into the legal system, serving as a valuable resource for both forensic practitioners and the legal community [100]. The legal professionals include lawyers, attorneys, solicitors, and judges [101], and the legal system relies heavily on expert evidence to resolve complex factual questions, particularly in areas that require scientific explanations and must be presented in simple terms to a judge or jury [100, 102, 103]. Forensic science experts, specifically, apply knowledge and methodology from various scientific disciplines to resolve legal issues [100]. The fundamental role of an expert witness is to use their experience, knowledge, and training to provide the court with an unbiased assessment of clinical or specialized issues [103].

Furthermore, legal professionals are increasingly recognized as being vulnerable to secondary trauma due to their repeated exposure to traumatic material and clients who have experienced trauma [101, 104, 105]. Secondary trauma is an umbrella term that includes vicarious trauma (VT), STS, compassion fatigue (CF), and burnout, all of which can arise from indirect exposure to trauma [101]. Iversen and Robertson [101] conducted a systematic literature review, including studies from 2003 to 2019, to analyze the prevalence of stress and burnout among legal professionals. They concluded that nine of the 10 studies reported elevated STS in legal professionals, and all studies that explicitly assessed burnout reported substantial levels, especially where caseloads and trauma exposure were higher (e.g., immigration/criminal contexts).

Another study by Maguire and Byrne [106] conducted a comparative analysis between legal professionals and mental health workers and found that lawyers scored significantly higher on levels of depression, anxiety, and stress compared to mental health workers. Moreover, criminal lawyers were likely to experience secondary trauma than non‐criminal lawyers, and judges exhibited higher levels than barristers [105]. Additionally, continuous exposure to indirect trauma can lead lawyers to withdraw, reducing their effectiveness and working hours [104, 107].

### 2.12 Forensic toxicology

Forensic toxicology is the branch of science that examines how drugs and chemicals affect the human body, with its findings applied directly to legal investigations and medicolegal cases [2, 108, 109]. There are three main subfields in forensic toxicology: postmortem (death investigation), human performance, and forensic drug testing [2, 108, 110]. Postmortem forensic toxicology involves the analysis of various fluids and tissues obtained during an autopsy to help establish the cause and manner of intoxication or death [108, 109, 111, 112]. Furthermore, human performance forensic toxicology evaluates the role of foreign chemical substances in altering human behavior, especially with respect to driving under the influence of drugs or alcohol and drug‐facilitated criminal acts [110, 113]. Finally, forensic drug testing involves demonstrating prior use or abuse of specific drugs through the analysis of biological samples, typically in workplace settings, for employment screening purposes [108, 109, 110].

Forensic toxicologists play a crucial role in identifying and quantifying toxic substances, as well as interpreting their probable role in a case [108]. Forensic toxicologists may also be called upon to testify in court as expert witnesses to explain their analytical findings and interpretations in the courtroom [112]. However, they may face several challenges in providing accurate and reliable information for legal proceedings [114]. For example, drug concentrations can change significantly after death due to factors like postmortem redistribution (PMR), where drugs move from tissues into blood [115], contextual bias that can affect decision‐making [116, 117], or a lack of standards for detecting and quantifying the emergence of new psychoactive substances [114].

### 2.13 General forensic science

The General Section includes the forensic disciplines that do not fall within the narrower definitions of other AAFS sections, as well as newly emerging forensic specialties [2]. It includes both traditional and evolving domains, including accounting, archaeology, art/sculpting, death and crime scene investigation, to name a few [2].

The two disciplines that particularly highlight the section's breadth are medicolegal death investigation and forensic veterinary science [118]. Medicolegal death investigators (MDIs) are professionals crucial to death investigations, operating within both the criminal justice and public health systems [119]. MDIs are often the initial point of contact for law enforcement and are responsible for responding to the location of death to document and conduct the investigation [120], representing medicolegal jurisdictions and forensic pathologists [118]. They are also responsible for gathering forensic evidence directly from the body and safely handling any biological or hazardous materials found at the scene [118]. On the other hand, forensic veterinary science is related to the health and welfare of animals [121]. Their work involves recovering, identifying, and analyzing evidence connected to cases of animal cruelty, neglect, unlawful killing, mistreatment, or the illegal trade of animals and their parts [121, 122].

### 2.14 Current study

The goal of the current study was to examine the psychological well‐being, coping mechanisms, and barriers to help‐seeking behavior for forensic science professionals in the AAFS. Founded in 1948, AAFS is a professional organization that focuses on the application of forensic science to the legal field. This organization is composed of several committees with over 6000 members spanning 50 United States and 71 different countries. In addition, the AAFS hosts an Annual Scientific Conference, which features oral presentations, papers, workshops, and offers professional education credits to those in the forensic sciences.

In the AAFS, there are twelve recognized disciplines: anthropology, criminalistics, digital and multimedia sciences, engineering and applied sciences, forensic nursing science, general, jurisprudence, odontology, pathology/biology, psychiatry and behavioral science, questioned documents, and toxicology. However, we specifically chose not to analyze differences between forensic science professions (e.g., digital forensics vs. forensic anthropology) out of concerns that this would only facilitate stigma and stereotypes, or individuals could be re‐identified if certain variables were linked (e.g., section, sex, age, race). We also felt that not asking members to identify their forensic science section would help reduce social desirability bias and increase their trust in the anonymous survey. For this study, we explored gender differences and years of work experience in relation to psychological well‐being, coping mechanisms, and barriers to help‐seeking behavior for current AAFS members.

## 3 METHODS

### 3.1 Participants

A total of 819 responses were received for this study as part of the consented online survey. Since the focus of this research study is on active members of the AAFS, respondents who indicated they were not active members (*n* = 43) or who declined to answer this question (*n* = 8) were excluded from the analysis. Additionally, 150 participants who started the survey but did not complete it were also excluded from the analysis. Therefore, the final responses for statistical analysis included 618 respondents who were active AAFS members and had completed the full survey.

As shown in Table 1, the majority of respondents were Caucasian/White (*n* = 627, 85.3%), female (*n* = 431, 69.7%), and married (*n* = 410, 66.3%). Participants' ages ranged from 20 to 86 years (M = 46.15, SD = 14.25), with 54.4% (*n* = 336) being between the ages of 36 and 55 years. Finally, slightly over half of the sample (52.5%, *n* = 324) self‐reported working in the forensic sciences for 16 or more years.

**TABLE 1:** Sample demographics.

Variables	Total (*n* = 618)
Gender	
Male	172 (27.8)
Female	432 (69.7)
Non‐Binary	11 (1.8)
Missing	4 (0.6)
Age	
18–25	32 (5.2)
26–35	118 (19.1)
36–45	176 (28.5)
46–55	160 (25.9)
56 or older	132 (21.4)
Ethnicity	
Caucasian/White	527 (85.3)
Others	84 (13.6)
Missing	7 (1.1)
Marital status	
Single	160 (25.9)
Married, CL, CU	410 (66.3)
D, S, W	44 (7.1)
Missing	4 (0.6)
Agency	
City	62 (10.0)
County	108 (17.5)
State	147 (23.8)
Federal	37 (6.0)
NGO/private	63 (10.2)
Academic/education	131 (21.2)
None	70 (11.3)
Work experience	
<1	24 (3.9)
1–5 years	84 (13.6)
6–10 years	88 (14.2)
11–15 years	97 (15.7)
16–20 years	111 (18.0)
>20	213 (34.5)
Missing	1 (0.2)

*Note*: *N* = 618. “None” for Agency includes individuals who reported as not working with specific agencies (e.g., retired, students, self‐employed, etc.). Note any discrepancies in percentage due to rounding. Values represent frequencies with percentages in parentheses.

Abbreviations: CL, Common Law; CU, Civil Union; D, divorced; S, separated; W, widowed.

### 3.2 Measures

Participants completed an anonymous online survey designed to gather data on demographic characteristics, psychological well‐being, coping strategies, and behavioral health needs. The Behavioral Health Needs Assessment Survey (BHNAS) was adapted from the Naval Unit Behavioral Health Needs Assessment Survey (NUBHNAS; [123]) and comprised multiple items assessing psychological well‐being, perceived social support, and attitudes toward and experiences with mental health care, including barriers to access. The study protocol was approved by the authors' Institutional Review Board (IRB # 2025‐138).

### 3.3 Psychological health and well‐being

The NUBHNAS measured psychological distress using the Kessler Psychological Distress Scales (K10 and K6) [124]. K10 and K6 measured non‐specific psychological symptoms such as depressed mood (*α* = 0.93), anhedonia (*α* = 85), motor agitation (*α* = 0.89), motor retardation (*α* = 0.74), fatigue (*α* = 0.78), worthless guilt (*α* = 0.88), concentration (*α* = 0.80), death/suicide thoughts (*α* = 0.68), anxiety (*α* = 0.90), worry (*α* = 0.86), motor tension (*α* = 0.73), hypersensitivity (*α* = 0.84), vigilance (*α* = 0.89), and positive affect (*α* = 0.92). All Cronbach's alphas were acceptable. The scale also included items measuring changes in eating habits (eating less, eating more) and sleep (sleeping less, sleeping more). In the NUBHNAS, items were rated on a 1–6 Likert scale (1 = “Never” to 6 = “Always”), and the higher scores indicate greater non‐specific psychological distress [124].

In this study, the PTSD Checklist for DSM‐5 (PCL‐5; [125]) was used to evaluate the potential presence of STS among participants. The PCL‐5 is a standardized, self‐administered questionnaire consisting of 20 items that reflect common symptoms associated with STS. Example items include prompts such as, “Feeling distant or cut off from other people?” and “avoid thinking about or talking about a stressful case” Unlike the earlier PCL‐IV, which offered separate versions for military, civilian, and specific populations, the PCL‐5 uses a single format and a 5‐point scale ranging from 1 (“Not at all”) to 5 (“Extremely”). Based on the scoring guidelines, all item responses are summed for a total symptom severity score (ranging from 0 to 80). Based on the total symptom severity score, scores of 31 or greater are considered indicative of meeting the criteria for probable PTSD symptoms or greater PTSD symptom severity [125]. To ensure that responses reflected work‐related stress from exposure to disturbing evidence, the survey introduced the scale with the following context statement: “Your work involves evidence that can be extremely stressful. Because of your current forensic science role, how often in the past month have you experienced…” Cronbach's alpha for the PCL‐5 was *α* = 0.94, a value considered acceptable.

### 3.4 Coping mechanisms

In this study, the coping strategies used by participants, whether adaptive or maladaptive, were evaluated using a modified 16‐item coping scale [61]. The authors examined 19 items grouped into seven categories of coping mechanisms, as outlined in [61]. These items included: distraction/suppression (e.g., “I sleep more than usual…”), with six items (1–5 and 19); substance use (e.g., “I take a some form of drugs…”), with five items (6–10); talking with others (e.g., “I participate in some organized…”), with three items (11–13); withdrawal/escapism (e.g., “I just try to be alone…”), with one item (14); prayer or meditation (e.g., “I engage in some religious…”), with one item (15); problem‐solving (e.g., “I seek professional help…”), with one item (16); and eating habits (e.g., “I eat less…”), with two items (17–18). Item references are presented in Table 3. Responses were measured on a six‐point scale from 1 (“Never”) to 6 (“Always”), with higher scores indicating more frequent use of that coping strategy. The purpose of this scale was to capture the range of coping mechanisms participants used to manage work‐related stress while reviewing disturbing evidence.

### 3.5 Behavioral health needs assessment

Respondents completed 14 items from the Mental Health Advisory Team (MHAT) survey, designed to assess perceived barriers to and stigma surrounding mental health care [123]. Each item was rated on a 5‐point Likert scale ranging from 1 (“Strongly Disagree”) to 5 (“Strongly Agree”). The questions addressed factors that might influence participants' willingness to seek counseling, such as “It would harm my career,” or “I would be seen as weak.” The reliability of the 14‐item version of the survey was acceptable, with Cronbach's alpha of 0.92. Higher MHAT scores indicate greater perceived stigma, more concerns about seeking care, and stronger perceived barriers.

In addition, participants were asked to respond to eight questions designed to assess their attitudes toward counseling and treatment [21]. For each item, they indicated “Yes,” “No,” or “Indifferent” in response to questions, such as “do you believe counseling should be required for those employed in the forensic sciences and allied forensic…,” “do you think counseling would help improve your productivity at work,” or “have you ever sought counseling or treatment as a result of your work.”

### 3.6 Procedure

The internet‐based study was hosted on Qualtrics, and the participants were recruited via an email listserv and social media posts made by the AAFS. Given the sensitive nature of this study, data were collected through a fully anonymous, self‐reported online survey. No identifying information was gathered, which helped minimize bias and enhance the validity of participants' responses on socially sensitive topics [126]. As part of the eligibility requirements, participants were required to self‐report that they were at least 18 years old and were currently active members of the AAFS. The survey was completely voluntary; participants had the option of skipping any questions or declining to respond.

The participants who completed the online survey were redirected to a separate “Thank You” page, where they could provide their email address to be entered into a drawing pool; 20 participants were randomly selected by AAFS to receive a $50 Amazon e‐gift card as compensation for completing the survey. We reiterate that we utilized two different Qualtrics survey pages: one for collecting survey responses and another for collecting the email addresses of participants who opted to enroll in the compensation drawing. This separation ensured that respondents could not be linked to their survey data.

### 3.7 Analytical strategies

We used IBM SPSS Statistics V29 for all statistical analyses and set the alpha level at 0.05. The following continuous variables were created in accordance with their scoring guidelines: general psychological well‐being (e.g., depression), PTSD Sum, coping mechanisms, and mental health barriers. Next, we created a dichotomous variable for PTSD (No/Yes), indicating which individuals met the DSM‐5 criteria for probable PTSD symptoms based on the total sum score (i.e., scores ≥31 suggested greater PTSD symptom severity). For general psychological well‐being (e.g., anxiety) and coping mechanisms (e.g., going shopping), we created dichotomous variables by collapsing their Likert scale to reflect “Never to Sometimes” vs. “Often to Always” to indicate the number (*f*) of participants who reported experiencing symptoms or using certain coping mechanisms more often. In addition, we also collapsed the MHAT Likert scale to indicate “Do Not Agree,” “Neutral,” and “Agree” to reflect the number (*f*) of professionals who agreed that these stigmas would impact their decision to get treatment.

First, descriptive statistics (M, SD, *f*) explored the sample's self‐reported mean scores for general psychological well‐being (e.g., depression), coping mechanisms, and mental health barriers. Correlations examined the relationship between “number of years working in the forensic sciences” and psychological well‐being, coping mechanisms, and mental health barriers; in general, we interpreted correlations as an effect size, with clinical importance approaching 0.20 [127, 128]. In addition, *t*‐tests examined gender differences (Male/Female) for each of the variables of interest. Finally, a chi‐square test examined the relationship between PTSD (No/Yes) and gender (Male/Female).

## 4 RESULTS

### 4.1 Descriptives

Of the 618 forensic science professionals, 432 (70%) reported knowing someone who has sought counseling or treatment, with over one‐third of respondents (*n* = 240, 38.8%) reporting that they sought counseling due to work‐related stress. However, only 3.4% (*n* = 21) reported that counseling has been required for their job position. In addition, 3.7% (*n* = 23) reported that they were required to notify their employer if they sought therapy as a result of work‐related stress; interestingly, 102 (16.5%) of forensic science professionals said they were “unsure” if they were required to notify their employer.

When asked if they would attend counseling if it was offered but not required, 47.9% (*n* = 296) reported “Yes” and 20.2% (*n* = 125) reported “No.” When asked if they believe counseling should be required, 27.7% (*n* = 171) reported “Yes,” but 26.2% (*n* = 162) were “Indifferent,” and 46% (*n* = 284) said “No.” Finally, over a third of forensic science professionals (38.5%, *n* = 238) thought they currently needed counseling due to work‐related stress, and 42.1% (*n* = 260) thought counseling would improve their productivity at work. Of the 618 forensic science professionals, 601 completed the PCL‐5 questionnaire. PCL‐5 scores ranged from 0 (which was the lowest possible) to 69 (the highest possible score was 80), with an average score of 11.88 (SD = 12.00). Based on the cut‐off score, 8.4% (*n* = 52) of the 601 forensic science professionals scored within the range that suggests having probable PTSD symptoms. The Chi‐square test indicated no statistically significant group association between gender (Male/Female) and PTSD (No/Yes), as 8.9% of men (*n* = 15) and 8.4% of women (*n* = 35) in the sample met the criteria for probable PTSD symptoms. However, there was a statistically significant association between seeking therapy and having probable PTSD symptoms (No/Yes); 76.5% of the forensic science professionals who had probable PTSD symptoms self‐reported seeking therapy as a result of work‐related stress, *χ* ^2^(1, *N* = 599) = 32.77, *p* < 0.001, *ɸ* = 0.23.

### 4.2 General psychological well‐being and PTSD

As shown in Table 2, forensic science professionals self‐reported on average higher scores for experiencing fatigue (M = 3.42, SD = 1.10) and losing sleep (M = 3.15, SD = 1.41) as a result of work‐related stress. Thoughts of death (M = 1.56, SD = 0.82) and anhedonia (M = 1.78, SD = 1.00) were, on average, the least experienced psychological symptoms reported by forensic science professionals. Next, we also compared the number of forensic science professionals who scored “often to always” to those who “rarely or only sometimes” experienced psychological symptoms. Nearly one‐third of forensic science professionals reported “often to always” experiencing loss of sleep, followed by a quarter of professionals experiencing loss of concentration and hypervigilance. Finally, approximately 20%–23% of forensic professionals reported “often to always” experiencing depression, motor agitation, anxiety, or motor tension. Less than 5% of forensic science professionals reported “often to always” experiencing thoughts of death, and less than 10% reported problems with motor retardation, anhedonia, and hypersensitivity. Finally, the same proportion of respondents reported having a positive attitude “rarely to sometimes” as those who reported “often to always” (See Table 2).

**TABLE 2:** Descriptives for psychological well‐being and probable PTSD symptoms based on gender and working years of experience.

Variables	M (SD)	*f* (%) “never to sometimes”	*f* (%) “often to always”	Years working^c^	Gender^d^		
Psychological well‐being^a^					Male (*n* = 160)	Female (*n* = 160)	Comparison
Depression	2.38 (0.99)	490 (79)	127 (20.6)	−0.15***	2.08 (0.86)	2.46 (1.00)	*t* = −4.56, *p* < 0.001
Anhedonia	1.78 (1.00)	567 (91.7)	49 (7.9)	−0.15***	1.64 (0.94)	1.81 (0.99)	*t* = −1.89, *p* < 0.05
Motor agitation	2.41 (1.22)	479 (77.5)	135 (21.8)	−0.18***	2.08 (1.11)	2.51 (1.20)	*t* = −3.89, *p* < 0.001
Motor retardation	1.98 (0.94)	554 (89.6)	59 (9.5)	−0.03	1.79 (0.81)	2.06 (0.99)	*t* = −3.37, *p* < 0.001
Fatigue	3.42 (1.10)	271 (43.9)	346 (56)	−0.14***	3.80 (0.98)	3.55 (1.10)	*t* = −5.00, *p* < 0.001
Worthless guilt	1.91 (1.01)	544 (88)	74 (12)	−0.21***	1.64 (0.92)	1.97 (1.00)	*t* = −3.59, *p* < 0.001
Concentration	2.61 (1.09)	453 (73.3)	164 (26.5)	−0.16***	2.21 (0.89)	2.75 (1.10)	*t* = −6.04, *p* < 0.001
Death	1.56 (0.82)	586 (94.8)	30 (4.9)	−0.06	1.63 (0.87)	1.52 (0.77)	*t = 1.59, p = 0.056*
Anxiety	2.45 (1.01)	483 (77.3)	140 (22.7)	−0.20***	2.06 (0.86)	2.56 (0.99)	*t* = −6.00, *p* < 0.001
Worry	2.42 (1.09)	504 (81.6)	113 (18.3)	−0.20***	2.11 (0.89)	2.53 (1.12)	*t* = −4.66, *p* < 0.001
Motor tension	2.50 (1.12)	478 (77.3)	140 (22.7)	−0.15***	2.18 (1.08)	2.61 (1.13)	*t* = −4.06, *p* < 0.001
Hypersensitivity	2.01 (0.78)	557 (90.1)	60 (9.7)	−0.18***	1.83 (0.69)	2.07 (0.78)	*t* = −3.48, *p* < 0.001
Hypervigilance	2.53 (1.04)	459 (74.3)	156 (25.2)	−0.09*	2.18 (0.97)	2.65 (1.02)	*t* = −5.00, *p* < 0.001
Eating more	2.26 (1.06)	551 (89.2)	67 (10.8)	−0.07	2.05 (0.92)	2.33 (1.10)	*t* = −3.03, *p* = 0.001
Eating less	2.04 (0.99)	570 (92.2)	48 (7.8)	−0.15***	1.89 (0.88)	2.08 (1.02)	*t* = −2.15, *p* < 0.05
Sleeping more	2.09 (1.11)	546 (88.3)	69 (11.2)	−0.24***	1.86 (0.92)	2.19 (1.17)	*t* = −3.15, *p* < 0.001
Sleeping less	3.15 (1.41)	395 (63.9)	222 (35.9)	−0.05	2.57 (1.27)	3.33 (1.40)	*t* = −6.28, *p* < 0.001
Positive affect	3.45 (0.97)	307 (49.7)	308 (49.8)	0.05	3.62 (0.95)	3.41 (0.96)	*t* = 2.40, *p* < 0.01
	**M (SD)**	**Not symptomatic**	**Symptomatic**				
PTSD Sum^b^	11.88 (12.00)	549 (88.8)	52 (8.4)	−0.07	10.23 (12.18)	12.32 (11.48)	*t* = −1.92, *p* < 0.05

^a^ Likert scale ranged from 1 (Never) to 6 (Always).

^b^ Likert scale ranged from 0 (Not at all) to 4 (Extremely). Due to missing data, percentages may not add up to 100.

^c^ *Listwise N* = 579; ***p < 0.001, *p < 0.05.

^d^ *Listwise N* = 566; Values represent means with standard deviations in parentheses.

In general, Table 2 suggests that forensic science professionals with more work experience reported better psychological well‐being. Fewer years of work experience was significantly correlated with more symptoms of worthless guilt, anxiety, worry, hypersensitivity, motor agitation, and sleeping too much– suggesting moderate effect sizes. Smaller effect sizes were also found between fewer years of work experience and depression, anhedonia, fatigue, concentration, motor tension, hypervigilance, and eating less. Years of work experience were not significantly related to positive affect, PTSD, motor retardation, thoughts of death, eating more, or sleeping less.

There were statistically significant mean differences between gender and nearly all of the psychological well‐being variables. On average, there was a marginal difference in that men (M = 1.63, SD = 0.87) reported slightly more thoughts of death compared to women (M = 1.52, SD = 0.77). However, compared to women (M = 3.41, SD = 0.96), men reported higher mean scores for positive affect (M = 3.62, SD = 0.95). For all of the other psychological well‐being variables (e.g., depression, anxiety, fatigue, etc.), women scored on average significantly higher, suggesting they reported experiencing more symptoms compared to men (see Table 2). Women also had, on average, higher scores for probable PTSD symptoms compared to men (M = 12.32, SD = 11.48; M = 10.23, SD = 12.18, respectively).

### 4.3 Coping mechanisms

As shown in Table 3, forensic science professionals reported higher average scores for using distraction (e.g., working harder, finding another activity), talking with friends or family, and withdrawing from others as coping mechanisms. On average, the least reported coping mechanisms were using drugs, smoking, and taking a sedative. We also compared the number of forensic science professionals who self‐reported “often to always” versus those who “rarely or only sometimes” used certain coping mechanisms. 50.3% of forensic science professionals self‐reported “often to always” talking with their spouse as a form of coping mechanism, and nearly half used some form of distraction (i.e., working harder was 46.8% and finding another activity was 47.6%). Nearly a third of forensic science professionals reported “often to always” using withdrawal (31.4%) or trying to forget about it (31.9%) as coping mechanisms.

**TABLE 3:** Descriptives for coping mechanisms based on gender and working years of experience.

Coping mechanisms	M (SD)	*f* (%) “never to sometimes”	*f* (%) “often to always”	Years working^a^	Gender^b^		
					Male (*n* = 160)	Female (*n* = 406)	Comparison
I work harder than usual around the house or on the job	3.56 (1.27)	329 (53.2)	289 (46.8)	−0.06	3.30 (1.24)	3.67 (1.26)	*t* = −3.29, *p* < 0.001
I just try to forget about it	3.01 (1.26)	418 (67.6)	197 (31.9)	−0.03	3.05 (1.36)	3.00 (1.21)	*ns*
I find some activity to take my mind off of things, like going to a movie	3.52 (1.18)	323 (52.3)	294 (47.6)	−0.11**	3.48 (1.28)	3.52 (1.34)	*ns*
I sleep more than usual	2.62 (1.38)	469 (75.9)	148 (23.9)	−0.22	2.28 (1.204)	2.75 (1.37)	*t* = −4.15, *p* < 0.001
I go shopping	2.31 (1.51)	523 (84.6)	93 (15.0)	−0.16	1.83 (0.91)	2.50 (1.19)	*t* = −7.30, *p* < 0.001
I have a drink, such as beer, wine, or a cocktail	2.51 (1.30)	490 (79.3)	128 (20.7)	−0.01	2.36 (1.26)	2.56 (1.31)	*t* = −1.64, *p* = 0.05
I take some form of drug, such as marijuana	1.12 (0.54)	606 (98.1)	9 (1.5)	−0.04	1.16 (0.63)	1.10 (0.50)	*ns*
I take a sedative, such as a sleeping pill	1.46 (1.05)	588 (95.1)	30 (4.9)	0.06	1.34 (0.85)	1.49 (1.08)	*t* = −1.73, *p* < 0.05
I take some other form of medication, such as an anti‐anxiety pill	1.88 (1.62)	528 (85.4)	89 (14.4)	−0.08	1.45 (1.78)	2.04 (1.72)	*t* = −4.71, *p* < 0.001
I smoke more often	1.14 (0.64)	603 (97.6)	15 (2.4)	−0.09*	1.14 (0.55)	1.15 (0.68)	*ns*
I talk things over with my spouse or significant other	3.59 (1.56)	303 (49.0)	311 (50.3)	−0.04	3.42 (1.40)	3.69 (1.61)	*t* = −2.08, *p* < 0.05
I talk things over with my friends	3.35 (1.25)	364 (58.9)	252 (40.8)	−0.13**	2.96 (1.21)	3.50 (1.24)	*t* = −4.84, *p* < 0.001
I participate in some organized groups or clubs to get social support	2.21 (1.30)	519 (84.0)	98 (15.9)	0.07	2.33 (1.37)	2.14 (1.70)	*t = 1.61, p = 0.05*
I just try to be alone and away from everyone	3.12 (1.26)	423 (68.4)	194 (31.4)	−0.12**	2.85 (1.31)	3.22 (1.23)	*t* = −3.20, *p* < 0.001
I engage in some religious or spiritual activity	2.02 (1.38)	529 (85.6)	89 (14.4)	−0.11**	2.20 (1.43)	1.95 (1.37)	*t* = 1.97, *p* < 0.05
I seek professional help, such as counseling	2.17 (1.32)	530 (85.8)	86 (13.9)	−0.10*	1.91 (1.26)	2.26 (1.32)	*t* = −2.91, *p* < 0.01
I eat more than usual	2.85 (1.25)	449 (72.7)	169 (27.3)	−0.14***	2.45 (1.10)	3.02 (1.27)	*t* = −5.10, *p* < 0.001
I eat less than usual	2.28 (1.05)	550 (89.0)	66 (10.7)	−0.14***	2.09 (0.95)	2.34 (1.06)	*t* = −2.69, *p* < 0.01
I exercise more than usual	2.37 (1.17)	522 (84.5)	95 (15.4)	0.03	2.41 (1.91)	2.34 (1.16)	*ns*

*Note*: Likert scale ranged from 1 (Never) to 6 (Always); due to missing data, percentages may not add up to 100.

^a^ *Listwise N* = 600; ***p < 0.001, **p < 0.01, *p < 0.05.

^b^ *Listwise N* = 566; Values represent means with standard deviations in parentheses.

We found several moderate and small correlations between the number of years working in the forensic sciences and several coping mechanisms. Specifically, statistically significant moderate correlations suggested that those who had worked fewer years were more likely to use sleeping more (*r* = −0.22) and going shopping (*r* = −0.16) as coping mechanisms. In addition, statistically significant, but small, correlations also suggested those who worked fewer years were more likely to use: distraction (i.e., finding another activity; *r* = −0.11), smoking (*r* = −0.09), talking things over with friends (*r* = −0.13), withdrawing (*r* = −0.12), engaging with religion (*r* = −0.11), seeking counseling (*r* = −0.10), eating more (*r* = −0.14), and eating less (*r* = −0.14). Overall, forensic science professionals with fewer years on the job self‐reported using more coping mechanisms than those professionals with more years of experience.

Although mean scores were relatively similar, there were statistically significant differences between gender and various coping mechanisms (See Table 3). On average, women self‐reported engaging in the following coping mechanisms more than men: distraction (i.e., working harder), sleeping more, shopping, drinking alcohol, taking a sedative, taking medication, talking things over with family or friends, withdrawing seeking counseling, and eating more or less than usual. On the other hand, men reported higher scores on average for engaging in religious activities and participating in organized groups or clubs. Overall, women self‐reported engaging in more coping mechanisms than men.

### 4.4 Mental health barriers

As shown in Table 4, forensic science professionals self‐reported that, on average, they generally disagreed that these mental health barriers would affect their decision to receive mental health counseling or services. Approximately one‐third of forensic science professionals agreed that “it would be difficult to get an appointment” and “it would be difficult to get time off from work for treatment.” In addition, 18% of forensic science professionals agreed that “being seen as weak” or the fear that “superiors would treat them differently” were barriers toward help‐seeking. The least reported barriers that would affect their decision to receive counseling were “superiors discouraging the use of mental health services” or “not trusting mental health professionals.” Interestingly, a number of forensic science professionals self‐reported being indifferent regarding the impact of that barrier on help‐seeking behavior. Specifically, about a quarter of forensic science professionals reported being neutral on whether “it was difficult to get appointment” and “it being difficult to get to the location.” In addition, 17.5% and 18.3% of professionals reported being indifferent to “it might affect my promotion” and “not trusting mental health professionals” as possible barriers to help‐seeking, respectively.

**TABLE 4:** Descriptives for MHAT based on gender and working years of experience.

Mental health stigmas	M (SD)	*f* (%) do not agree	*f* (%) neutral	*f* (%) agree	Years working^a^	Gender^b^		
						Male (*n* = 169)	Female (*n* = 426)	Comparison
I don't trust mental health professionals	1.86 (0.92)	473 (76.5)	113 (18.3)	31 (5.0)	0.01	1.96 (1.03)	1.81 (0.87)	*t* = 1.85, *p* < 0.05
Mental health services aren't available	1.93 (0.100)	466 (75.4)	89 (14.4)	61 (9.9)	−0.10*	1.70 (0.87)	2.01 (1.03)	*t* = −3.47, *p* < 0.001
I don't know where to get help	1.94 (1.05)	474 (76.7)	60 (9.7)	83 (13.4)	−0.09*	1.82 (1.04)	2.00 (1.06)	*t* = −1.83, *p* < 0.05
It is difficult to get an appointment	2.68 (1.26)	274 (44.3)	157 (25.4)	184 (29.8)	−0.11**	2.49 (1.22)	2.77 (1.25)	*t* = −2.48, *p* < 0.01
There would be difficulty getting time off work for treatment	2.54 (1.10)	345 (55.8)	83 (13.4)	188 (30.4)	−0.20***	2.11 (1.29)	2.73 (1.41)	*t* = −5.15, *p* < 0.001
It's too difficult to get to the location where the mental health specialist is	2.21 (1.12)	378 (61.2)	150 (24.3)	88 (14.2)	−0.05	1.98 (1.01)	2.31 (1.16)	*t* = −3.42, *p* < 0.001
It would be embarrassing	2.05 (1.13)	423 (68.4)	103 (16.7)	90 (14.6)	0.01	2.09 (1.09)	2.07 (1.15)	*ns*
It would harm my career	1.90 (1.06)	467 (75.6)	95 (15.4)	55 (8.9)	−0.05	1.89 (1.10)	1.91 (1.05)	*ns*
Members of my unit/department might have less confidence in me	1.98 (1.11)	442 (71.5)	93 (15.0)	81 (13.1)	−0.02	1.93 (1.10)	2.01 (1.12)	*ns*
My superiors might treat me differently	2.16 (1.23)	405 (65.5)	97 (15.7)	114 (18.4)	−0.03	2.09 (1.22)	2.19 (1.24)	*ns*
My superiors would blame me for the problem	1.93 (1.12)	461 (74.6)	84 (13.6)	71 (11.5)	−0.01	1.83 (1.02)	1.96 (1.15)	*ns*
I would be seen as weak	2.10 (1.17)	426 (68.9)	79 (12.8)	112 (18.1)	−0.02	2.09 (1.09)	2.12 (1.20)	*ns*
It might affect my promotion	2.04 (1.16)	428 (69.3)	108 (17.5)	80 (12.9)	−0.05	1.98 (1.13)	2.05 (1.16)	*ns*
My superiors discourage the use of mental health services	1.68 (0.87)	497 (80.4)	98 (15.9)	20 (3.2)	−0.03	1.74 (1.00)	1.65 (0.86)	*ns*

*Note*: Likert scale ranged from 1 (Strongly Disagree) to 5 (Strongly Agree); due to missing data, percentages may not add up to 100.

^a^ *Listwise N* = 600; ***p < 0.001, **p < 0.01, *p < 0.05.

^b^ *Listwise N* = 595; Values represent means with standard deviations in parentheses.

There was a statistically significant correlation between years of work experience and four mental health barriers (see Table 4). Specifically, a moderate effect size (*r* = −0.20) suggested forensic science professionals with fewer years of work experience were more likely to agree that “it would be difficult to get time off from work.” Statistically significant, although smaller correlations, indicated those with fewer years of work experience were also more likely to report that “mental health services are not available” (*r* = −0.10), “they do not know where to get help” (*r* = −0.09), and “it is difficult to get an appointment” (*r* = −0.11). There was no statistically significant relationship between years of experience and the other mental health barriers.

In general, both men and women reported average scores toward the lower end of the Likert scale, suggesting that they disagreed with these barriers impacting their help‐seeking behavior (see Table 4). However, there were significant mean differences between gender and six of the 14 barriers to help‐seeking behavior. On average, women self‐reported higher scores for the following barriers compared to men: “mental health services are not available;” “don't know where to get help;” “it's difficult to make an appointment;” “it would be difficult to take time off from work;” and “it is difficult to get to the location.” Men, on average, only reported significantly higher scores for “not trusting mental health professionals” compared to women.

## 5 DISCUSSION

Several studies have found that forensic science professionals experience psychological distress due to exposure to challenging and high‐stakes work environments [8, 9, 10, 12, 13]. However, there is a lack of literature on the psychological well‐being and mental health needs assessment of forensic science professionals, especially for AAFS forensic professionals. Therefore, the goal of this study was to analyze the psychological well‐being, coping mechanisms, and mental health barriers experienced by members of AAFS in general, as well as differences based on gender and years of work experience.

We found that forensic science professionals reported higher scores on experiencing fatigue and sleeping less than usual, as compared to the other psychological well‐being symptoms. Moreover, sleeping less than usual (36%) was more commonly endorsed at the ‘often to always’ level, followed by concentration (26.5%) and hypervigilance (25.2%). This finding is consistent with Holt et al. [129], who reported that nearly 30% of forensic scientists experienced sleep difficulties, over 15% reported having difficulty concentrating, and approximately 12% reported being easily startled. The work in the forensic field keeps the professionals physiologically “switched on,” with irregular or on‐call schedules (e.g., crime scene investigators; [15]), distressing graphic material (digital evidence examiners/investigators; [21, 61]), and getting ready for court (legal professional; [101]). We also found that depression, motor agitation, anxiety, and motor tension were also endorsed at the ‘often to always’ level by approximately one‐fifth of the forensic science professionals, which highlights a significant mental health burden within these professions. This is consistent with broader research indicating that forensic professionals, depending on specific fields, are at increased risk for experiencing psychological distress [129, 130, 131]. For example, medical examiner and coroner (ME/C) office employees, who include investigators, administrators, and forensic laboratory personnel, have been found to report significant levels of anxiety and depression [6, 71, 120].

Our findings also indicate that forensic science professionals with fewer years of work experience were more likely to report psychological distress symptoms (e.g., worthless guilt, anxiety, worry, hypersensitivity, motor agitation, etc.), which is consistent with findings reported by Holt et al. [129]. Stress tends to decrease with more experience, suggesting that time in role brings procedural confidence, better control over schedules, and more effective coping strategies [129]. Early‐career examiners may face a steep learning curve when handling graphic material and tight deadlines [9, 132]. However, Almazrouei et al. [15] highlighted that forensic examiners with 11–15 years of experience reported significantly higher stress from workplace factors than those with 7–10 years of experience. For example, for first responders, a category that encompasses specific forensic roles, approximately one in four (25%) reported suffering from depression as a consequence of their work, with more experienced personnel being more affected [133]. Moreover, for legal professionals, there are inconsistent findings; some studies highlighted that more years on the job did not affect psychological well‐being [101, 134, 135], and others mentioned that more time in the job role was linked to higher STS [101, 136].

Based on the gender, men were more likely to report suicidal ideation and positive affect as compared to women. However, women scored significantly higher for all the other (16 out of 18) psychological well‐being symptoms, including depression, anxiety, fatigue, etc. It broadly aligns with the Almazrouei et al. [15], which highlighted that female forensic examiners experience more general and workplace stress than their male counterparts. Moreover, research indicates that female forensic professionals in demanding professions, such as criminal justice [14, 129, 133] and healthcare roles [17, 137], often face a greater psychological burden. This increased vulnerability for women may be due to occupational stressors such as gender discrimination [20], lack of support from male counterparts [20], and harassment [133] in male‐dominated environments. Furthermore, we found that women were more likely to have probable PTSD symptoms as compared to men, which is in contrast with the findings of [10]. Although women had significantly higher PCL‐5 scores, both men and women were well below the clinical cut‐off score. Moreover, female participants in this study reported higher psychological distress and PCL‐5 symptom severity as compared to men. However, these gender differences may be due to differential reporting tendencies rather than true differences in psychological burden. For instance, a study conducted by Ko and Memon [4] found a greater effect of STS in women but mentioned that it may be because women are more likely to admit and report symptoms of STS than men. In addition, differences may exist in how mental health symptoms manifest in response to stress, with women more likely to experience internalizing disorders (e.g., depression) and men more likely to experience externalizing disorders (e.g., substance abuse) [138, 139]. Overall, gender differences in mental health disorders are likely impacted by how these disorders manifest and are reported.

For coping mechanisms on the “often to always” level, our study indicates that forensic science professionals primarily reported positive coping strategies (e.g., talking with their spouse or friends). In contrast, less than 5% reported the use of maladaptive strategies (e.g., drugs). These findings align with the study conducted by Holt et al. [129], which suggests that forensic professionals are more likely to employ positive coping strategies than maladaptive ones. However, more people reported distraction as a coping mechanism, which may be viewed as negative, as it often leads to emotional suppression [18] and ignoring problems, which can have negative long‐term consequences [20]. The high percentage of professionals (50.3%, “often to always”) reported talking with their spouse or with friends/family, which underscores the critical role of social support. This “approach‐based strategy” is widely recognized as beneficial for mental health [12, 140]. However, some forensic practitioners, including crime scene investigators and police staff working with sexual offense materials, hesitate to share traumatic details at home, either to protect loved ones or because of self‐stigma [129].

When comparing the coping mechanisms used based on work experience years, forensic professionals with fewer years of work experience were more likely to employ positive coping strategies, except for a few maladaptive ones (e.g., smoking), compared to those with more work experience. This may be because more experienced professionals are well aware of their role and are comfortable with their work [14]. Additionally, the likelihood of less experienced forensic professionals using more coping strategies may be due to their higher initial vulnerability to occupational stressors [9]. Finally, when comparing coping mechanisms used based on gender, women were more likely to use more coping strategies than men (11 out of 19). This may be due to female examiners experiencing a higher level of general stress (such as family responsibilities) and workplace stress compared to male examiners, thus reflecting inherent differences in coping strategies [15].

The results also indicate that there are different barriers related to mental health services for forensic science professionals. They were more likely to agree with 4 out of 14 mental health barriers. In addition, one‐fourth of the forensic science professionals were neutral regarding the mental health‐seeking barriers due to the accessibility of appointments, and approximately one‐fifth were neutral about affecting the promotion and not trusting mental health professionals.

When comparing mental health barriers with years of work experience, the individuals with fewer years of work experience were more likely to agree with barriers toward mental health services due to availability. Goldstein and Alesbury [8] highlight that the common mental health issues are often overlooked during workplace training, academic journeys, and certification processes. This lack of knowledge about accessing resources highlights the importance of increasing awareness and education about mental health and the process of seeking support during onboarding for new employees [141]. Therefore, introducing concepts of wellness and VT into academic schooling and early career stages is seen as crucial for better preparation [8]. These training programs should incorporate techniques for managing stress, fostering resilience, and developing practical coping strategies [19].

Finally, on comparing mental health barriers based on gender, women were more likely to report mental health barriers (5 out of 14), whereas men reported 1 out of 14. Women reported challenges with the availability of resources, challenges with their time off, and a lack of awareness. Men reported “not trusting mental health professionals” as the barrier to mental health. Women often handle additional household and childcare responsibilities, which may create work–family conflict and stress, making it challenging for them to take time off from work for appointments [9, 15]. Therefore, forensic organizations should invest in promoting stress‐management strategies, recognizing that these contribute to a psychologically healthy workforce [20].

Moreover, it is also important to provide information on organizational resources (e.g., employee assistance programs and insurance‐approved providers) to facilitate individualized assistance [133]. It is essential for management to actively promote awareness of available mental health services among forensic scientists, ensuring that access is clearly communicated and encouraged in a supportive, non‐stigmatizing manner [129]. Additionally, supportive organizational policies play a crucial role, including those that promote flexible work arrangements, conflict‐free roles, and effective communication between employees and their supervisors [10].

### 5.1 Limitations and future research

This study provides an analysis of well‐being among forensic science professionals; however, it has limitations. First, we did not ask participants to report their specific section within the AAFS community to maintain anonymity and reduce social desirability bias; therefore, the results provide a general overview of psychological distress and mental health barriers among all forensic science professionals. Some sections may experience more psychological distress and mental health barriers than others, depending on the nature of the work they are involved in. Secondly, we did not ask for exposure details (e.g., field vs. lab duties), frequency/severity of disturbing evidence, shift patterns, or caseloads. The role demands and content severity are known to shape STS and burnout [10, 142].

More research is needed to identify effective strategies to strengthen organizational culture to reduce mental health stigma and normalize help‐seeking behavior for female forensic professionals. Additionally, understanding robust ways to encourage early‐career and female forensic science professionals to access mental health services regularly is essential. It is important to acknowledge the higher stress levels, vulnerability, and availability/awareness barriers for mental health services among females in examiner roles [8, 15]. Future research is needed to identify which wellness policies and awareness initiatives effectively strengthen resilience and reduce work‐related stress among forensic science professionals, especially for females and early‐career professionals. Wellness policies also need to explicitly address gender discrimination and sexual harassment, which may be significant stressors for women [133]. The wellness programs may include protected time for mental health appointments [129], peer network support [10, 20], mentorship for females and early‐career professionals, and stress management [20].

## 6 CONCLUSION

In conclusion, the current study addresses a critical gap by analyzing the mental health needs of AAFS community practitioners by examining general psychological well‐being, PTSD, coping mechanisms, and mental health barriers. The authors also analyzed these behaviors based on years of working experience and gender. The authors conclude that, based on gender, women are more likely to report most of the psychological distress as compared to men. In addition, forensic science professionals with fewer years of experience were more likely to report psychological distress. Similarly, the study highlights that female forensic science professionals and forensic science professionals with fewer years of experience reported using more coping strategies and experienced more mental health barriers. Overall, our findings support the importance of implementing a supportive organizational culture, including training, awareness programs, and encouraging help‐seeking behaviors, irrespective of gender and years of experience. These efforts are essential to addressing the psychological challenges and stigma faced by forensic science professionals dealing with work‐related stress.

## FUNDING INFORMATION

We are grateful to the 2025 AAFS President and Academy leadership for supporting the Ad Hoc Vicarious Trauma Committee and providing incentive funding for this project.

## CONFLICT OF INTEREST STATEMENT

The authors have no conflicts of interest to declare.

## Data Availability

The data that support the findings of this study are available from the corresponding author upon reasonable request.
